# Self-* Capabilities of Cloud-Edge Nodes: A Research Review

**DOI:** 10.3390/s23062931

**Published:** 2023-03-08

**Authors:** Raúl S-Julián, Ignacio Lacalle, Rafael Vaño, Fernando Boronat, Carlos E. Palau

**Affiliations:** Communications Department, Universitat Politècnica de València (UPV), 46022 Valencia, Spain

**Keywords:** self-* capabilities, heterogeneous nodes, computing continuum, computational nodes, edge-cloud nodes, Internet of Things, review

## Abstract

Most recent edge and fog computing architectures aim at pushing cloud-native traits at the edge of the network, reducing latency, power consumption, and network overhead, allowing operations to be performed close to data sources. To manage these architectures in an autonomous way, systems that materialize in specific computing nodes must deploy self-* capabilities minimizing human intervention across the continuum of computing equipment. Nowadays, a systematic classification of such capabilities is missing, as well as an analysis on how those can be implemented. For a system owner in a continuum deployment, there is not a main reference publication to consult to determine what capabilities do exist and which are the sources to rely on. In this article, a literature review is conducted to analyze the self-* capabilities needed to achieve a self-* equipped nature in truly autonomous systems. The article aims to shed light on a potential uniting taxonomy in this heterogeneous field. In addition, the results provided include conclusions on why those aspects are too heterogeneously tackled, depend hugely on specific cases, and shed light on why there is not a clear reference architecture to guide on the matter of which traits to equip the nodes with.

## 1. Introduction

The concept of the “cloud” appeared in the telecommunications world about thirty years ago, in the 1990s, when the first Virtual Private Networks (VPN) began to be used. Cloud computing (CC) appeared sometime later as a new way of computing, seeking to offer scalable virtual environments to meet the new needs of users [1].

Today, CC is one of the most widespread and used methods for performing complex calculations that require a large number of computing cycles or for the analysis and processing of large amounts of data that require the highest possible speed of execution. Additionally, it is considered one of the most important changes in the field of Information Technology (IT) for society [2]. In 2011, the National Institute of Standards and Technology (NIST) of the United States Department of Commerce defined CC as a model for enabling anywhere, convenient, on-demand network access to a set of shared and configurable computing resources (servers, storage, services, etc.) that can be provided and released quickly and with very little effort. This model is mainly composed of three service models: Software as a Service (SaaS), Platform as a Service (PaaS), and Infrastructure as a Service (IaaS) [3].

CC architecture consists of a large network of servers from different providers, distributed worldwide and connected to the internet, capable of running large workloads or making services available to users for free or for a fee. There are three types of cloud models: public, private, and hybrid [4]. These servers began by organizing themselves in small cores that grew and are interconnected exponentially over time to form a complex network. The purpose of CC is the efficient combination of distributed resources to perform tasks that require large computing power or offer services [4]. The scalability, the large amount of data that it is capable of processing, and the practically unlimited processing and execution capacity of calculations are some of the characteristics that make CC a valid solution for most cases [5].

However, despite the great computing and data storage capacity of this paradigm, CC presents several shortcomings that are difficult to solve for certain situations. The aforementioned great work capacity requires big computing centers that are generally far away from the source of data generation. Among many others, this produces some disadvantages such as high latency and low response times, as the information has to travel through many points across the network [5]. These drawbacks prevent calculations and data processing in real time, with low response times, far from the source of information. Moreover, these data centers consume a lot of energy, generating a huge carbon footprint. This high and inefficient energy consumption has become a big problem today, partially rooted in the lack of exploitation of renewable energies to feed such big centers [6]. In fact, depending on the geographical area, the energy mix may vary. In countries where emissions regulations are tougher, renewable energies predominate. This means that part of the energy consumed by these data centers comes from renewable energy sources, reducing the carbon footprint generated. However, in countries with laxer or non-existent emissions regulations, fossil energy is usually the predominant one in the energy mix, which implies a greater environmental impact in its use.

In order to carry out these operations in real time, with very low latency and greater security in the transfer of information, the edge computing paradigm was created. This new way of working with information was empowered by the traits of the expansion of the Internet of Things (IoT), allowing calculations and data processing to be performed on nodes at the edge of the network, rather than on the cloud [7]. The idea was to reduce the energy impact and response times without noticing too much of a decrease in global capacity based on less powerful computing equipment at the edge of the network (closer to the data sources). In this way, all the information that was produced in the edge nodes was also processed within them. This should make it possible to reduce the workload of the data centers, avoiding network congestion and reducing the execution time of the time-sensitive applications [8].

However, this paradigm shift is not without challenges, starting from its definition as a concept. There are currently several ways to define edge computing. The Edge Computing Consortium defines it as an open, distributed platform at the edge of the network, close to data sources and integrating computing and data storage capabilities [9]. For Zhang et al. [10], edge computing is a novel form of computing that allows the storage and processing of resources near the source of the data, providing intelligent services that collaborate with CC. Shi et al. [8] define edge computing as enabling technologies that allow computations to be performed at the edge of the network, in the proximity of data sources. Not only do these nodes consume data, but they also produce and process data.

All in all, the advent of edge computing, in addition to fog computing, enabled the expansion of the so-called computing continuum to the edge of the network [11]. In order to control the small sets of IoT devices, alongside other equipment that globally comprise the edge computing system, autonomous systems -that do not require human intervention to function or to resolve errors that can occur- are needed. Thanks to the great evolution of the learning systems, such as reinforcement learning or deep learning [12], these intelligent systems are increasingly autonomous and require less human intervention.

To devise such autonomy, the elements that form the continuum (or computing fabric)—from now on, “nodes”—must embed certain self-* capabilities that allow for their independence of use as intelligent components in the network [13]. Self-* naming is adopted (and used across this work) due to its own capacity to realize certain characteristics that actually make them intelligent and independent. Over time, the number of self-* capabilities has been noticeable. Whereas many of them are essential in making a whole edge computing environment autonomous, others might be deemed dispensable, depending on the specific field of application.

This work mainly focuses on the exploration of the different self-* capabilities of existing autonomous intelligent systems and on the selection of those that are considered essential to declare an edge computing intelligent system “autonomous”. An analysis of the most relevant practical use cases currently available is carried out for each self-* capability. This detailed study of the self-* capabilities allows for the depiction of an up-to-date global vision of this research field. From a research perspective, this paper can be devised as a comprehensive review of the self-* capabilities of heterogeneous nodes of the continuum. However, especially most recently in the edge-cloud field, sources such as open-source development projects, public code repositories, blogs, or websites are being increasingly used for innovation reporting rather than delivering formalized scientific publications. This article covers a very wide spectrum (self-* capabilities); therefore, it has dug deep into the literature, adjusting the efforts to deliver usable information that could be later leveraged in further works.

This paper is organized as follows. In Section 2, the analysis of the context and the compelling need for such a review is carried out. In Section 3, a description of the methodology used and a discussion of the obtained results are provided. In Section 4, the classification of nodes in the computing continuum is described, and the definition of basic concepts is presented. In Section 5, the literature review and a comparative analysis of self-* capabilities are developed. Finally, in Section 6, the conclusions of the work carried out are drawn.

## 2. Background

While many reports and works [14] have concluded that CC is essential to digital transformation and digitalization for companies’ competitiveness, it is an undeniable truth that trends in the computing field of distributed systems pass through moving computation and intelligence to the edge of the network. Apart from some technical disadvantages associated with CC that have been outlined in Section 1, CC seems to go against the democratization of the computing industry [15], which should pursue a model of geographically dispersed “local grids” of lower-cost small-cloud capacity nodes (grasping the true potential of edge-cloud computing [16]). In addition, recent market studies estimate that the global edge computing market size will reach EUR 1.352 M by 2025 and EUR 7.013 M by 2028, responding to an average Compound Annual Growth Rate (CAGR) of 36.2% in that period.

According to the strategic agenda of relevant entities, such as the European Commission (EC), more data processing and decision making must be shifted to the edge to obtain competitive, smarter systems worldwide [17]). Here, reports such as H-CLOUD’s whitepaper [14], EAIDCE [18], FCC [19], and EAA&BI [20] coincide in pointing to the edge-to-cloud hybrid paradigm as a strategic technology towards leadership in the digital space [21]. Advances in this field might help the industry at large to maintain and establish the full control of data flows, and how they are processed and stored, from the edge to high-density clouds, providing on-demand, secure data conduits supporting full user autonomy and fostering the applicability of data sovereignty initiatives. This impact could be affected by the high centralization of cloud infrastructures and by the move of the so-called exa-scalers to occupy the edge space. Many sectors will benefit from a proper implementation of the continuum, leveraging more intelligence delegated into heterogeneous nodes, such as energy infrastructure (smart grid, renewable energies, electric vehicle charging stations, etc.), national security (maritime ports, cybersecurity, logistics, traceability of goods, etc.), or healthcare (personalized dynamic treatment per patient, monitoring, pharmaceutical supply chain, etc.), among others [22].

As a matter of fact, the EC has launched an ambitious initiative: EUCloudEdgeIoT [23], which aims at bringing together all European research actions seeking to devise the computing continuum with a common stack of open-source technologies. This is aligned with the increasing presence of the continuum and its computing elements in official texts such as those for funding tenders, including the quest for meta-operating systems to be installed in heterogeneous nodes [24] or the smart orchestration of the computing fabric leveraging Artificial Intelligence (AI) [25].

This is very relevant for understanding the background of this article. The authors heavily rely on their experience in research actions funded by the EC. Realizing the previous facts, this work aims at paving the way for a reference architecture of the self-* capabilities of nodes in the computing continuum, building upon the European principles of openness and governance.

In particular, the context of this work has roots in the research guided by relevant European-funded actions. On the one hand, the project ASSIST-IoT is an ongoing initiative that intends to devise the reference architecture for the next-generation IoT deployments. Building on top of cloud-native principles and the usage of orchestrated Kubernetes(K8s)-like distributions, diverse computing elements (clusters) are controlled to deliver advanced IoT characteristics and services. Here, leveraging K8s-assimilable nodes that can be heterogeneous equipment in the continuum opened the door for implementing certain self-* capabilities. Concretely, that project has advanced the comprehension of a system as an intelligent entity, including the self-awareness and semi-autonomous behavior of services (containers) deployed across the network. In addition, it has served the authors in understanding the field of action (self-* capabilities in a distributed environment), leading to the devising of the content of this article. On the other hand, the project aerOS aims at delivering a meta-operating system for governing the computing continuum. Here, a special focus is put on the Infrastructure Elements (IEs) that compose such a continuum. The IEs are actually heterogeneous computing nodes that include IoT devices, smart components, network elements with execution capacity, personal computers, micro servers, data centers, etc. One of the key aspects of achieving the governance of the continuum is allowing these nodes to be more aware (in a distributed, decentralized way) of their surrounding environment such that more capacities in the edge open up. It is in the context of this action that the authors of this work considered it necessary to conduct comprehensive research on the specific field.

Some works have been found addressing similar topics from a survey/review perspective; however, none of them adjusted to the above-mentioned scope. In [26], a taxonomy describing the continuum as an evolution of IoT and dynamic resources is proposed, as well as the different components related to cloud-native principles and edge paradigm fitting technologies. In that very work, the need for future viability studies on particular aspects of the continuum is called for. On another note, [27] digs deep into a survey of optimal application placement over the cloud-to-thing continuum, which can be considered a self-* quality of automated systems including heterogeneous nodes. It helps to categorize the issues in application placement in micro-services deployment through an inspiring review methodology but does not examine other self-characteristics. Additionally, the review [28] goes over diverse concepts that are very useful for achieving self-capabilities in the target scope of this work. First, autonomic computing lays the foundations for the self, and in that review, a deep analysis of closed and open-loop systems is performed. Second, AI promises to be a key element in (almost) any self-characteristic, as monitoring and inference will allow for introducing intelligence to the various nodes. However, that review only provides a global overview of those concepts, without explicitly tackling the wide spectrum of autonomous features, and it also covers topics further away from the work in this article, such as quantum computing. All in all, it is feasible to conduct a review, as proposed in this article, going beyond the current coverage to fill the gap in the self-capabilities of heterogeneous nodes of the computing continuum.

## 3. Research Methodology

A review of the literature on the different self-* capabilities was conducted to obtain an up-to-date vision of research and practical examples to understand the status of the field.

In order to carry out a precise search and to be able to discover and analyze the largest number of research works across the available literature, a method consisting of three steps has been followed. First, different sources of information have been selected to obtain the necessary works for the review. IEEE Xplore has been the main database used, complemented by other sources, such as the ResearchGate and ScienceDirect databases. Second, in-depth iterative searches have been carried out, combining the different keywords for each selected self-* capability in order to obtain more precise results that are close to the established search criteria. For each self-* capability, results have been obtained that made it possible to obtain definitions and practical use cases. In addition, from the searches carried out, those works of the greatest interest with the following characteristics have been selected:

- Written in English.
- Preference was given to those works published between 2015 and 2023. Although, due to their relevance, some works published previously have also been selected.

Mainly containing the keywords “cloud computing”, “edge computing”, “heterogeneous nodes”, “computing continuum”, “IoT”, “self-awareness”, “self-orchestration”, “self-diagnose”, “self-healing”, “self-scaling”, “self-configuration”, “self-optimization”, “self-adaptation”, and “self-learning”.

In addition, the review also included those articles of interest referenced in the works selected in the main search, looping into an iterative, cross-referenced approach. The last step consisted of analyzing all the selected articles and synthesizing the most important information from each one.

As a by-product of the conducted review, the authors also propose a taxonomy and terminology for the research in the field, which is depicted in Section 4. This was carried out after observing the inconsistency in the terms used across different papers. According to the authors, this is a consequence of the lack of reference articles tackling the narrow field of the self-* capabilities of heterogeneous computing nodes from a comprehensive perspective. Many works focus on one aspect or another, but an exercise of holistically analyzing the characteristics that make a wide edge computing system “autonomous” had not been carried out yet. More details on this reflection are provided in Section 4 and Section 6.

### Results

**Figure 1:**
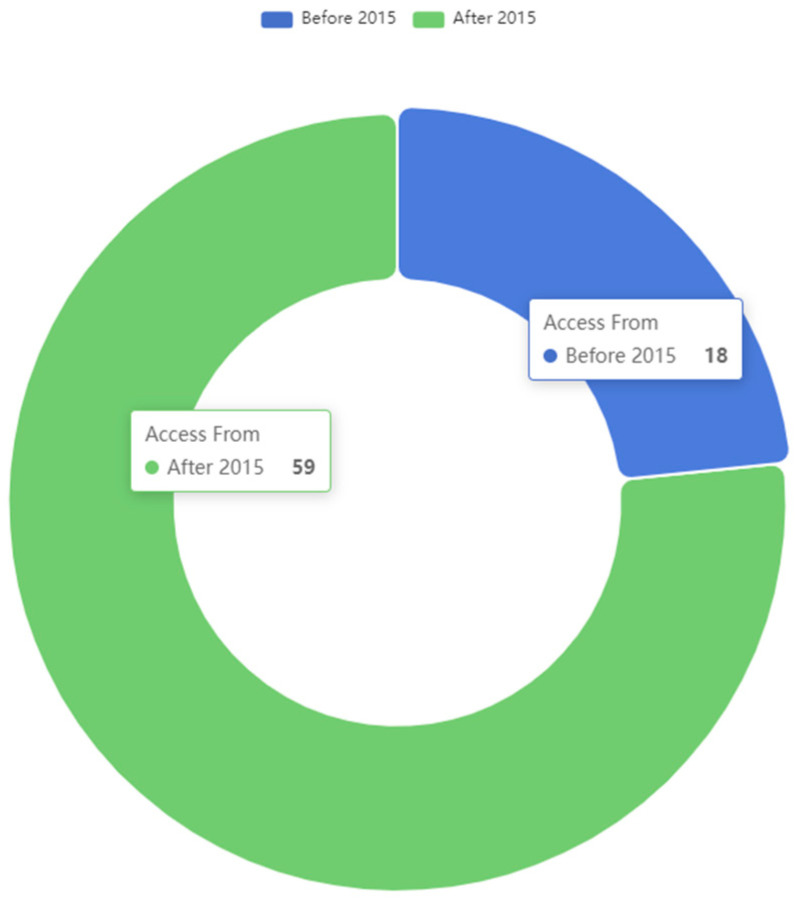
Number of publications before and after 2015.

As a result of the search, a total of 77 papers have been selected. A total of 24% of them (18 papers) were published before 2015. The remaining 76% (59 papers) were published between 2015 and 2023, as it can be seen in Figure 1. This is a relevant milestone to be highlighted, as it is considered that edge computing, as a used concept in the field of IoT and the continuum, was born in late 2015.

**Figure 2:**
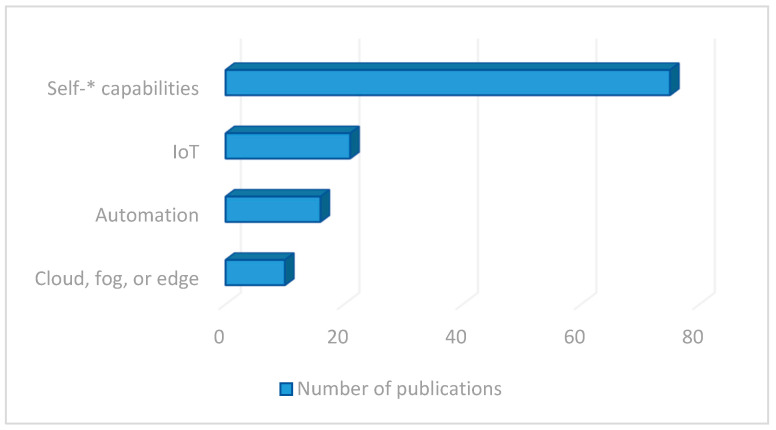
Number of publications by topic.

The fact that, approximately, three-quarters of the research articles referenced in the review date from after 2015 is a clear indication of the interest in intelligent and autonomous edge computing systems and nodes and the need for their use in certain fields (especially since the advent of practical edge and fog computing systems). As will be seen later, almost all implementations found in the literature are developments adapted to specific use cases, lacking any sort of “reference architecture” in the field. By analyzing the selected publications based on the keywords that appear in the titles or in the abstract or understanding the main themes that they develop (as depicted in Figure 2), several conclusions are obtained. There are 10 articles covering cloud, fog, or edge-related topics that include any self-related capability. A total of 16 papers thoroughly describe aspects related to automation. A total of 21 articles are directly related to the IoT world. A total of 75 papers contain in their title or abstract one of the self-* capabilities selected for the study.

**Figure 3:**
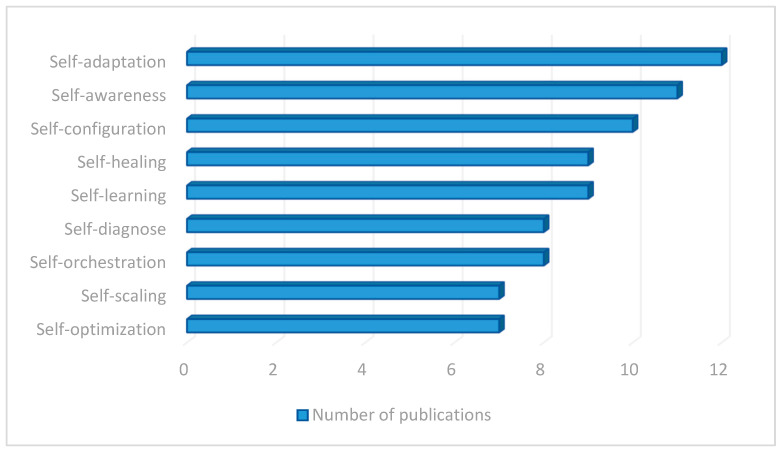
Number of publications by self-* capability.

A total of seventy-five (75) articles were analyzed that referred directly to one (or more) self-* capability out of those described above, as drilled down in Figure 3. It was observed that self-adaptation is the most investigated trait, with 12 devoted papers in total. This is followed by self-awareness, with 11; self-configuration, with 10; self-healing and self-learning, with 9; self-diagnose and self-orchestration, with 8 items; and self-optimization and self-scaling, with 7 items. The average number of articles per self-* capability is nine. It is worth mentioning that those papers focus almost exclusively on the indicated single feature (leaving aside or keeping marginal the others).

This detailed analysis of the research articles found in the search related to self-* capabilities provides an overview of which capabilities are the most referenced (and therefore necessary) when developing an intelligent and autonomous edge computing system composed of heterogeneous nodes. Self-adaptation, self-awareness, self-learning, self-configuration, and self-healing could be considered part of the most basic pillars of these systems.

## 4. Terminology and Taxonomy

**Figure 4:**
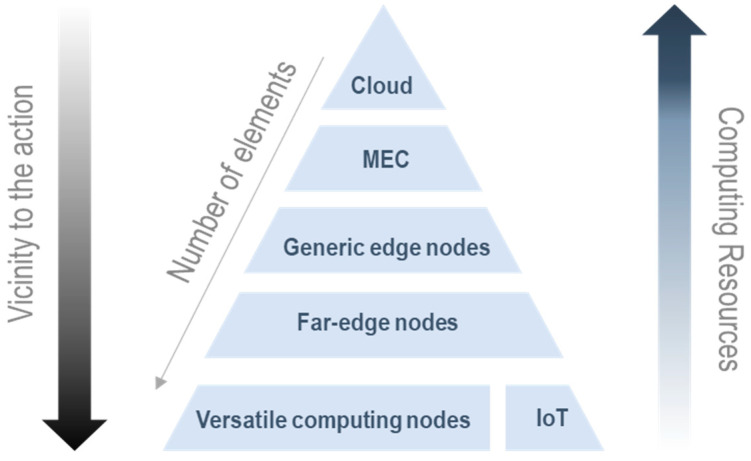
Classification of the heterogeneous nodes according to their spot on the continuum.

The computing continuum (also called the digital continuum, IoT-edge-cloud continuum [29], computing fabric, or transcontinuum) is the combination of resources and services at the center of the network (cloud), at its border (edge), and in transit (fog). Data are generated and pre-processed at the edge, partially processed by intermediate nodes, and, if necessary, transferred to the cloud [30]. A node is a physical (or virtualized) device that is part of a network and has the capability to execute certain computations and communicate with other nodes. Today, there is a wide variety of nodes that can connect to the continuum. All these nodes have different characteristics and architectures that make them unique. For this reason, it is appropriate to refer to them as “heterogeneous nodes”. There are several ways to classify them, depending on their architecture, type, location in the network, etc. Drawing from the nature of this work, a primary classification option for these heterogeneous nodes has been carried out according to their spot on the continuum (an illustrative diagram is provided in Figure 4). Within each category, a variety of capacities, features, powers, sizes, and specific characteristics also exist:

- Cloud nodes: high-performance servers and high-capacity storage systems that provide services to their users. They allow complex calculations to be executed and are capable of permanently storing a large amount of data [31]. Topologically, these are normally placed on a central location (data center).
- MEC (Mobile or Multi-Access Edge Computing) nodes: smart nodes, normally IT servers tied to radiocommunications infrastructure (e.g., in base stations [32]), that enable the capabilities of cloud services closer to the users’ devices (namely, smartphones or end terminals).
- Edge nodes: any device with computing, storage, and network-attached capabilities, which are capable of dividing and distributing large amounts of work. Examples of these devices are access points, routers, small servers, computers, base stations, etc. [33].
- Far-edge nodes: hardware devices capable of running algorithms that collect and pre-process information received from IoT devices or versatile computing nodes [34].
- Versatile computing nodes: geographically distributed physical devices closer to the end user such as commercial devices, such as Raspberry Pis, SIEMENS SIMATIC edge elements, personal computers, laptops, smartphones, tablets, wearables, smart cards, smart vehicles, etc., with enough computing power to execute tasks [31]. Versatile computing nodes can sometimes also be considered far-edge nodes; they are very close terms that vary mainly in their topological and geographical position, as well as in their role in an edge computing distributed system.
- IoT nodes: physical devices such as sensors, readers, surveillance cameras, actuators, embedded devices, etc. They are able to detect events or characteristics of real objects and transmit them to the upper layer for processing [5,31]. In most recent deployments, IoT nodes are increasingly improving their embedded computing capabilities, starting to act as versatile computing nodes. These are known as smart devices and are a genuine part of the Next-Generation IoT [35].

To reduce the response time, the security risks associated with the cloud, and the computing limitations of end nodes, MEC and Edge nodes have been proposed to address these and other related issues. However, MEC nodes have a much higher computing capacity than edge nodes [36]. For this reason, MEC nodes are commonly used to replace cloud nodes in the heaviest tasks.

As mentioned, a computing continuum is a combination of heterogeneous nodes that can connect with each other and cooperate, forming a network (or fabric). This involves a myriad of challenges in terms of virtualization and orchestration (see Section 2). Several works discussing this heterogeneity have been analyzed.

On the one hand, Razzaque et al. [37] comment that one of the main characteristics of these nodes is the heterogeneity, as mentioned above. On the other hand, Xiao et al. [38] state that this heterogeneity of the nodes makes their configuration more varied and their physical conditions more complex and changing, making their orchestration difficult. Although every node might be, potentially, running its own architecture, it is necessary to ensure that services are always executed regardless of the underlying configuration. This system not only has to be able to connect these nodes with the edge computing continuum, but it also must be able to manage them automatically so that each and every one of them has an autonomy of use. There is a current quest for searching for such a tool; several works and research projects are pursuing this goal [39].

Cluster computing and grid computing are other forms of computing that exist today. According to [40], cluster computing is a form of computing in which two or more computing nodes are connected in a local network to offer certain computing capacities or services to users. On the other hand, ref. [40] defines grid computing as a form of computing in which two or more hyper-distributed computing nodes are interconnected in the same network aiming to combine their resources to execute calculations that require many computing cycles. On another note, ref. [41] refers to the capability of self-deciding to offload tasks within a cooperative network of nodes in a vehicular computing continuum. Concepts such as fog colonies have appeared to describe the self-controlled, context-aware grouping of heterogeneous nodes by sharing contextual information and policy rulebooks in a decentralized approach [42].

The goal of this work is not to review the characteristics and self-* capabilities of the nodes of these forms of computing but rather to explore the different self-* capabilities needed in autonomous intelligent systems that are part of the computing continuum and to select those that are considered essential to declare a computing node ”autonomous”.

While holistic governance and orchestration are under investigation, this paper focuses on the relevance of looking at the capacity of the nodes to apply certain features to help this automation materialize. To obtain a true global intelligent continuum system, there is the claim that more intelligent and independent computing nodes must be achieved. This is what the authors depend on to devise the so-called self-* capabilities that must be intrinsically offered by such nodes. A self-* capability is a property of a heterogeneous node that, together with other basic self-* capabilities, allows it to operate independently, without the intervention of the upper layers of the continuum. According to the literature, there is a wide variety of self-* capabilities, organized and named in different ways depending on the chosen criteria. For instance, in [43], IBM explains that the essence of an autonomous system is self-management. Drawing from this statement, the four main aspects of self-management would be:

- Self-configuration: autonomous systems are capable of configuring themselves and their components, following high-level policies.
- Self-optimization: the capacity to continually improve their performance by monitoring and identifying their resources to become more efficient.
- Self-healing: automatic diagnosis and resolution of hardware and software faults.
- Self-protection: the ability to anticipate and avoid problems and autonomously defend against external attacks or internal failures with self-healing measures.

Berns et al. [44] define a more complete list of self-* capabilities, which are: self-management, self-stabilization, self-healing, self-organization, self-protection, self-optimization, self-configuration, and self-scaling. They also include two self-* capabilities from their own understanding:

- Self-immunity: the system is capable of restoring security predicates after an attack, eventually preventing them from being compromised again.
- Self-containment: the ability to keep functional parts of the system uncompromised by a malicious attack.

Sterritt et al. [45] expose a list of self-* capabilities by completing the one in [44] with the following: self-anticipating, self-assembling, self-awareness, self-chop, self-critical, self-defining, self-governing, self-installing, self-reflecting, self-similar, self-simulation, and selfware.

**Figure 5:**
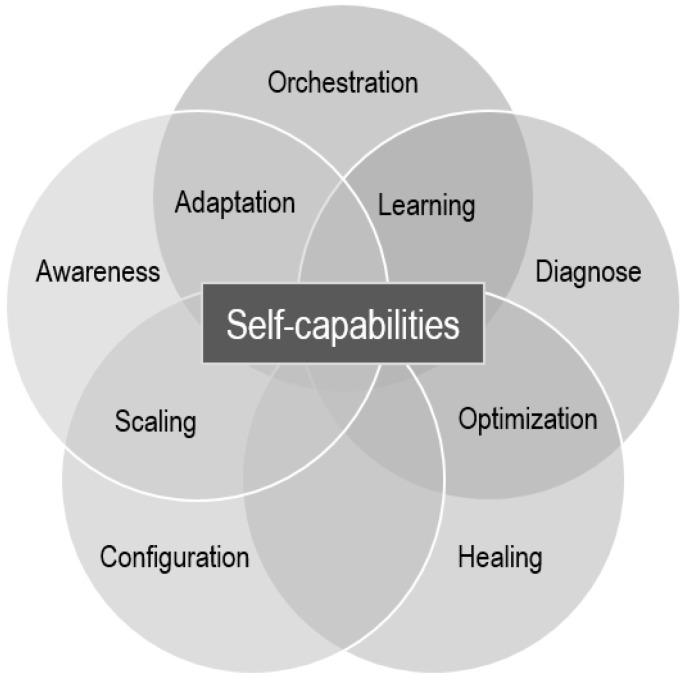
Classification of the self-capabilities defined for this article.

For this review, based on the previous references and the authors’ experiences in several research projects in the IoT, edge, and CC fields, it was decided to select the following self-* capabilities, reflected as well in the Venn diagram of Figure 5:

- Self-awareness.
- Self-orchestration.
- Self-diagnose.
- Self-healing.
- Self-scaling.
- Self-configuration.
- Self-optimization.
- Self-adaptation.
- Self-learning.

The practical application of these self-* capabilities should allow for autonomy of use and the awareness of the environment. If this is achieved, the global management of heterogeneous nodes towards an orchestration of the whole continuum would be hugely facilitated.

## 5. Literature Review and Analysis

This section provides the main review corpus of the paper. First, it describes what every self-* capability means according to the authors, based on the literature and their own research. Second, it delves into the investigation of each self-* capability in the available sources. The main goal has been to identify the constructive elements and concepts of the self-* features as well as to recognize any tools or methods used to materialize them. Finally, a comparison considering the depth and abundance of work per trait is provided.

### 5.1. Sensors and Systems of Sensors Overview

IoT sensors are devices that collect data from the surrounding environment (temperature, humidity, movement, etc.) to send it to the upper layers of the systems in which they are integrated and that can be processed for further analysis and interpretation. In sensor networks or systems, self-* capabilities are also used to control and automate the IoT devices (sensors) that comprise them.

In [46], Yeh et al. propose a fault self-diagnosis technique for sensor networks based on FBG (Fiber Bragg Grating). With this technique, when a network or sensor failure occurs, the exact location can be detected. Zhu et al. [47] developed a self-diagnosis and self-detection system for integrated sensor networks capable of receiving and processing information from the environment. This system predicts the data captured by the sensors in real time and compares them with the real data to determine the accuracy of the data acquisition, that is, the correct operation of the sensors. Furthermore, if a sensor failure is detected, the system can diagnose the cause. Richardson and Cheneler [48] present a set of ideas for the self-diagnosis, self-adaptation, and self-healing of autonomous sensors integrated into electronic systems through software algorithms. The objective is to increase the reliability of the data generated by the sensors and allow them to repair themselves, emulating the resilience of living beings. Bicocchi et al. [49] present a framework for carrying out unsupervised training between sensors in the same network. The objective is to exchange information between the sensors so that they learn a model using the data obtained by other sensors. To do this, they use, as a use case, the combination of a camera and an accelerometer to identify the movements of the users.

### 5.2. Analysis of Self-* Capabilities Research Status

#### 5.2.1. Self-Awareness

Götzinger et al. [50] define self-awareness as an ability of computer systems to observe and analyze the environment surrounding them and themselves, with the aim of making changes in their behavior, according to the observations made. They also comment that self-awareness is the base in an autonomous system for all other self-* capabilities. In [51], the authors explain that self-aware computing systems need to gain knowledge about the controlled resources and their environment. This knowledge can be extracted from the analysis of the execution time of tasks, employing machine learning (ML) algorithms over internal and external data or from other sources. In systems with hierarchical architectures, knowledge can be affected due to the loss of a part between higher and lower levels. This is the case of the computing continuum as understood in this work. Although the goal is to conceive all available resources as a single entity to be managed, geographically and topologically, each node is constrained to its direct visibility, living in an inner hierarchical layout. Esterle and Brown [52] state that the nodes of a network must be aware of other systems and devices further away from their immediate environment.

Articles [52,53] propose five levels of self-awareness of connected systems that have access to network resources and network monitoring parameters (such as the performance of different model-building algorithms, the objectives of other systems, trade-offs between targets, etc.):

- Networked stimulus-awareness: allows the system to know how to respond to events in its environment with the stimuli received.
- Networked interaction-awareness: determines that the stimuli received and the actions performed form relationships with the surrounding environment.
- Networked time-awareness: obtains information about historical stimuli in order to predict future stimuli and their effect on other nodes.
- Networked goal-awareness: having knowledge of the objectives, goals, constraints, and preferences of the rest of the nodes allows them to know how it affects them, based on specific tables dependent on network information.
- Networked meta-self-awareness: the system is capable of determining its own level of network self-awareness and how it is exercised.

In [54], Anzanpour et al. propose a monitoring and control system for the health of hospital patients with a self-aware design. This system is based on wearable devices (with limitations such as power consumption or performance) that obtain data through sensors such as heart rate, blood oxygen, blood pressure, or body temperature. This information is sent to cloud servers for their storage and processing. This system provides personalized care, self-organization, and autonomy of use for remote monitoring and intelligent decision making based on the situation for patients. Here, the principles of self-awareness are delegated to the cloud, pulling away from the edge computing nodes (sensors and smartphones); however, the mechanisms still apply for a potential self-awareness system design. Andrade and Torres [55] propose a conceptual model of cognitive security, with self-awareness as the main element. Here, a computer system (potentially assimilable to a heterogeneous node) is capable of generating learning models (based on self-aware knowledge) and reasoning models (created from the defined learning models).

Approaching self-awareness and control formalization, IBM [56] proposed a feedback loop for autonomic control called “MAPE-K”. This model has five phases:

- Monitor: obtain data and information from the environment for the node self-awareness.
- Analyze: the most important information obtained in the monitoring phase is selected and studied.
- Plan: the necessary actions for achieving goals and objectives are defined and built.
- Execute: the procedures for the execution of the plans are defined.
- Knowledge: the information used in the four previous phases is stored as shared knowledge.

Any self-awareness methodology or tool to be embedded in a heterogeneous node in the continuum should consider this methodological approach in its design.

In [57], Elhabbash et al. proposed a generic system that uses symbiotic simulation to address the difficulty of analyzing the quality of knowledge and achieving the capacity of meta-self-awareness of the system, which allows it to know its levels of consciousness. Ref. [58] introduced a framework, based on the analysis and extension of three bio-inspired theories, for descriptive and generative dynamic models that strengthen the capacity of self-awareness of autonomous systems. The three bio-inspired theories are the models of Damasio, Haykin and Friston et al., and Zhang et al. [59], who discuss cognitive digital twins, examine the concepts of digital twins and self-awareness together, and explore the possibility of harnessing different levels of self-awareness for cognitive digital twin design.

In summary, self-awareness has been tackled in the literature from three main perspectives: (1) the formal methodology -MAPE-K-, defining the steps to be performed to feed a system back towards such awareness; (2) based on the network to understand its own needs and act upon the context and a series of objectives; and (3) from a list of specific cases, mostly related to health applications, without going further and placing awareness on heterogeneous nodes of the continuum.

#### 5.2.2. Self-Orchestration

The synchronous and sequential execution of services is called orchestration. Orchestration systems include the application logic needed to manage services [60]. This is one of the most important capabilities in distributed systems because it allows applications to meet the requirements of end users in a specific order, and associated complexity is managed by proper internal components. Moreover, it improves the scalability of applications and minimizes failures between inner modules [61]. Based on the definition of orchestration in [62], the authors adopt the definition of self-orchestration as the self-* capability of nodes to configure themselves, manage themselves, and coordinate with each other to achieve common goals and objectives. That is, it focuses on the architecture of the system, the applications that compose it, and the data they manage with the requirements of the business.

In [60], Delamer and Lastra describe the difficulties in providing rapid reconfigurability in current and future manufacturing systems in the industrial sector. Based on this, the authors analyze the concepts and definitions of self-orchestration and choreography oriented to web services at the node level and propose the use of self-orchestrated semantic web services to solve the problem. Khebbeb et al. [62] present a rewriting-based specification developed in Maude to design and verify the self-adaptive and orchestration behaviors of the cloud and fog layers in order to manage the reconfiguration of the architecture and manage the self-adaptation and orchestration of the cloud and fog layers based on a centralized control pattern to achieve low latency and resources quantity trade-offs.

The authors of the paper [63] propose a new reference for Building Automation Systems (BAS). This paradigm is heavily inspired by social network interrelationship models for improving the self-configuration and self-orchestration of nodes in the home and smart building automation. The developed framework is based on social objects and semantic descriptions of resources and services. This increases the autonomy of the use of the devices, their capabilities to configure themselves, and the relationship between them and the environment that surrounds them. These devices take on the role of intelligent agents, which can self-configure, self-coordinate, and self-orchestrate. The proposed model was implemented on Arduino boards and on Intel Edison and Zolertia single-board computers with more resources.

In [64], Schulz focuses on the development of a model whose objective is to define the self-management and self-organization of a network as if it were a subsystem within automation systems. In this way, all components of the communication architecture are defined, implemented, and maintained in an automated manner. The model is applied to Intranets within companies at an industrial level, orchestrating the transport of information through IP and legacy protocols as well as wired and wireless connections interchangeably. The author intends that the developed model serve as a reference for other research and as a standard in IoT networks at an industrial level.

Regarding self-orchestration, it can be concluded that there is not a common understanding of which kind of self-orchestration can be achieved. However, there are several documented attempts to orchestrate inner networks and the use of their own resources in the form of intelligent agents.

#### 5.2.3. Self-Diagnose

Self-diagnosis is the self-* capability of a smart node or device to continuously monitor its health status [65]. The node has the ability to detect the error and its origin, which allows for the development of highly reliable and energy-efficient applications [66]. However, the term self-diagnosis is also applied to networks made up of intelligent nodes that are capable of self-diagnosis or of sending their health status to central nodes for further analysis. Examples of these networks can be found in [67,68,69,70].

Discenzo et al. [65] evaluated the need for IoT devices for the self-diagnosis of components in the industry. Thanks to a small engine together with a microprocessor, they developed a model for self-diagnosing its status and preventing possible future failures. In [67], the author addresses the development of “Promising”, a model capable of self-diagnosing the state of a network and its nodes. The method is based on the use of a highly reliable checking component to evaluate the state of the nodes of a network. In addition, the author recommends monitoring in a decentralized manner to minimize network traffic.

Rahem et al. [68] describe possible failures that can occur in data aggregation. This technique is commonly used to analyze and diagnose the status of Wireless Sensor Networks (WSN) due to their low power and bandwidth consumption, reduced execution time, etc. In this work, in addition, an analysis is carried out on the data added by the central node in the cluster to evaluate the energy consumption, using self-diagnosis. This node manages all the operations and devices that compose the controlled group. In [69], Harte et al. also develop a model to monitor the health status of nodes within a WSN using self-diagnosis. The authors focus primarily on detecting physical problems in devices caused by impacts or them not being properly oriented.

In order to identify failures and errors in ad hoc mobile networks and wireless mesh networks, the authors of [70] proposed a novel self-diagnosis model called “Adaptive-DSDP”. This protocol is based on comparison, where tasks are assigned to pairs of nodes, and the results obtained are analyzed and compared.

As it can be realized, self-diagnosis is tackled in the literature not as a characteristic of the nodes themselves but as part of a group or a network. Whether this is due to a lack of usage of the term self-diagnosis or to the dismission of its own health diagnostic, the reality is that, for modern edge computing environments, this aspect will need to be thoroughly tackled in the near future.

**Figure 6:**
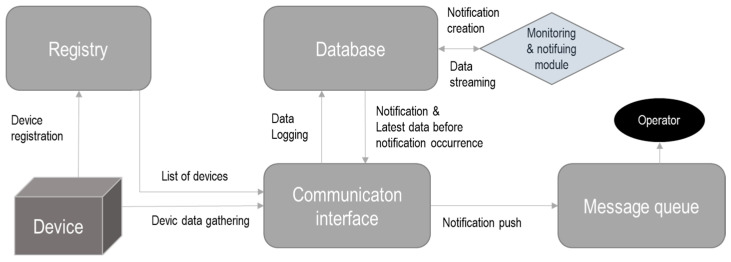
Self-monitoring approach, as proposed by ASSIST-IoT. Source: the authors’ own diagram, based on [73].

In the ASSIST-IoT project, a modular software was developed (see Figure 6) that allows for the self-monitoring of edge device functionalities, logs, etc. and the generation of notifications if a failure occurs. This module is part of the self-* vertical plane enablers [71,72].

#### 5.2.4. Self-Healing

Self-healing is the part of autonomous systems that is responsible for independently managing the recovery of the parties affected by a failure or attack without human intervention. This mechanism provides the ability to maintain and resume the system in an automatically set condition [74]. Khalil et al. [75] also include failure detection as part of self-healing. In [43], IBM explains that self-healing is the self-* capability to automatically diagnose and resolve both hardware and software failures.

Yang et al. [76] developed and implemented a self-healing system for the electrical network made up of several Easergy T300 controllers installed in medium-voltage feeders (20.000 V) that monitor the state of the electrical network through an analysis and self-healing algorithm in real-time to detect failures and avoid prolonged power outages. The controllers analyze the load of the feeders, obtaining data on the temperature of the devices, energy, etc. in order to manage the network. Thanks to the self-healing algorithm, the system is capable of identifying the type of fault and its location, isolating the sector of the network with problems and reconfiguring the network to re-energize the areas affected by the fault. In this way, the duration of power outages can be reduced from hours to just seconds autonomously. The methodological principles of this work are very relevant for a potential shift of the self-reparation to heterogeneous computing nodes instead of high-voltage network controllers.

In [77], the authors also develop an autonomous control system for the monitoring and self-healing of the smart distribution network based on distribution automation and advanced distribution automation. The self-healing of the system includes preventive self-healing, fault self-healing, and economical self-healing. This intelligent system is able to adapt to the complex environment formed by these networks, continuously monitoring and managing resources. Thanks to this, the system is able to ensure and improve the electrical supply of the network in the event of a problem thanks to the use of resources such as power generators widely distributed throughout the network, energy storage devices, and even electric vehicles connected to the network (V2G).

The control of autonomic systems, through monitoring their health status, is one of the essential parts of self-healing algorithms, which is connected to the self-diagnose capability treated above. Other works [74] propose a monitor model that can improve the self-healing performance by decreasing the number of resources spent on the self-healing-affected parts of the system.

There are works that propose using Neural Networks (NN) to avoid failures in distributed computing systems. In particular, there are self-healing algorithms that are based on replacing defective hardware nodes, which cause system overloads [75], with new ones. NNs are complex algorithms used in a wide variety of applications [75], especially in the field of AI. From another viewpoint, Khalil et al. [75] propose a novel method to be applied to self-healing NNs; using a single node per layer, it is possible to replace any defective node. If a node fails, its neighbor will also perform its tasks (apart from those already assigned to it) sequentially. If the neighboring node fails, only the spare node will take over, reducing the load on the system.

Liu et al. [78] show the design and implementation of a zero-time self-healing communication network for real-time ship monitoring. This network is capable of connecting sensors, control devices, and computers to interact with the ship’s maintenance team. Through various control and surveillance mechanisms, it is capable of automating many of the tasks carried out on ships. The objective of this novel design is to solve the transmission, reliability, and real-time problems of network communications. To do this, it transmits the information through several routes to have a seamless and instantaneous self-healing network. Thanks to this network, the maintenance of the ship becomes easier and faster.

In [79], as in [76,77], the author exposes a model for the automatic reconstruction of the electrical network with self-healing capacity to avoid power outages to users and reduce the cost of repairing the electricity network.

In the case of the self-healing capability, the majority of the found literature focuses on the electrical power of devices (either computing or not) or the electrical distribution network. Some works also explore the self-healing of computing nodes that can be part of a continuum. However, there are only a few mentions of the self-recovery of the communications or the functioning of single nodes—for instance, when the connection is down or faulty.

**Figure 7:**
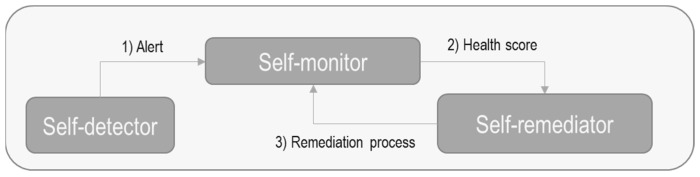
Self-healing structure, as proposed by ASSIST-IoT. Source: the authors’ own diagram, based on [73].

In ASSIST-IoT, a self-healing modular software (as per the diagram in Figure 7) was developed in order to recover from failures to the IoT devices that incorporate it. This recovery is based on an already existing schedule of routines. This enabler is divided into three components:

- Self-detector: its purpose is to obtain information from the device on which it works.
- Self-monitor: check the health status of the IoT device, analyzing the information obtained by the self-detector component. From these data, health score metrics are extracted, which are compared with thresholds to determine if the device is OK or not.
- Self-remediator: if the self-monitor component detects a bad state of health of the device, it sends a notification to this component to try to recover (through a series of operations) the good state of health. If this is not possible, other operations are applied to try to recover the state of health again.

This module is part of the self-* horizontal plane enablers [72,80].

#### 5.2.5. Self-Scaling

Based on the definition offered by Herbst et al. [81] on scalability, the authors define self-scaling as the self-* capability of an intelligent node to increase or decrease the use of its resources depending on the volume of work to be carried out. If the workload increases, the node is able to increase its resource usage automatically. Otherwise, it will remove part of its resources to accommodate the volume of incoming work.

Herrera and Moltó [82] introduce two novel biology-inspired algorithms that enable self-scaling in architectures based on the execution of self-managed containers. The algorithms described are:

- Self-scaling self-sufficient cell model (SCM): this model is characterized by the lack of direct interactions between containers. This design, in turn, is subdivided into three variants (SCM-A, SCM-B, and SCM-C).
- Self-scaling interactive cell model (ICM): this model is characterized by containers that have information about the containers that are in their environment. The exchange of information can be carried out directly (between containers) or through intermediate services.

In [83], the authors describe a model for self-scaling the resources of a network based on the task execution times of each instance of virtual network functions (VNF). The resources used by each instance (both physical and virtual) are assigned per cycle unit using a weighting factor. The system is made up of two components: a self-scaling application (which includes several control and management modules) and a monitoring module based on micro-services.

Nikravesh et al. [84] propose an architecture for a self-scaling prediction ensemble based on empirical studies, which is capable of selecting the best prediction algorithm based on the amount of real-time workload.

Casalicchio and Perciballi [85] present a self-scaling algorithm called “KHPA-A” that connects to the Kubernetes controller and is based on absolute metrics rather than relative metrics. The use of this type of metric allows the system to reduce the response time of the applications compared to the current K8s self-scaling algorithm. In addition, this algorithm can make use of the input parameters used by the original “KHPA” algorithms to obtain the number of containers to be instantiated. Similar to this approach, in the research project ASSIST-IoT, an improved alternative to the Horizontal Pod Autoscaler (HPA) of Kubernetes based on time series inference (rooting on Facebook Prophet NN) and custom logic is proposed and developed: the “resource provisioning enabler” [72,86]. This software is able to horizontally scale (up or down) the resources devoted to a specific enabler (custom software packaged as a Helm chart and following a set of predefined encapsulation principles) within a K8s node in a dynamic fashion [87].

Chattopadhyay et al. [88] propose a self-scaling orchestration model for IoT applications called “Aloe”. This framework dynamically deploys lightweight controller instances close to IoT devices (which are resource-constrained) to ensure a high availability and low set-up time. It is fault-tolerant, can migrate instances from one site to another in cases of problems with part of the network, and uses Docker as a base to support migration.

As a reflection, self-scaling is the capability that has been brought to practice more frequently and successfully, mostly due to its capacity of making use of already existent tools provided by container management frameworks, as well as other applications.

#### 5.2.6. Self-Configuration

According to [89], the self-configuration of an application or autonomous system is the self-* capability to configure and reconfigure itself automatically and independently in any type of possible condition. In [43], IBM explains that self-configuration is the self-* capability of autonomous systems to configure themselves and their components, following high-level policies.

Yang et al. [90] developed a model to self-configure connected terminals in 4G networks and heterogeneous communication and service environments. When a terminal connects to the network, the framework puts it in pre-operational mode until the node self-configures, at which point the node becomes operational within the network. When a terminal leaves the network, the TMS (Terminal Management System) notifies the rest of the nodes so that they are aware of the new state and reconfigure themselves appropriately. Wang and Vanninen [91] describe and compare different protocols for individual peers to self-configure the P2P network. To determine which is the best protocol, they simulate small-scale P2P networks and compare the quality of self-configured networks.

Mombello et al. [92] presented a self-configuring system for a photodetector sensor. Its goal is to use a control unit that can be programmed to find the center of the light beam hitting the sensor and then set the detection pattern. This model allows for the automation of the alignment of the light beam with the detection pattern. For this, the model is capable of obtaining data from the light sensor to reprogram the behavior of the photodetector sensor in real-time. In [93], the authors describe a self-configuration algorithm for a modular robotic system (MRS). This system is made up of robots which move through a virtual grid until they reach their optimal position in the configuration space. Through local communications, the robots can analyze and plan routes within the grid to change position.

One of the advantages of self-configuration for heterogeneous nodes would be to facilitate better CI/D of services in computing elements at the edge of the network. Currently, as occurs with millions of serve-providing applications, updates on firmware and software versions must be carried out offline, requiring disconnection or rebooting of the components, including hardware. Achieving self-configuration in real time would smooth these processes that usually underserve the requesting users, which can be highly inconvenient in specific applications. Abdellaoui et al. [94] propose a real-time self-configuration system that is capable of automatically connecting and disconnecting the modules (components) that make up the applications to reduce service outages and cause the least possible interruptions. Each connected object in the application is considered as a software module that is added or removed to be updated separately. This could be a nice approach towards the self-configuration of heterogeneous nodes in the continuum.

Yao et al. [95] designed a system that automates the self-configuration of the use of virtualized shared resources in graphics cards of cloud servers intended for cloud-gaming. This framework is made up of four modules:

- Sensor module: gathers preliminary system and application data.
- Modeling module: automatically analyzes raw data from the sensor module.
- Controller module: for each virtual machine running on the graphics card, an agent monitors its performance and sends the information to a scheduler. This analyzes the information of all the virtual machines and sends an instruction to activate the control system.
- Self-control-configuration module: manages the self-configuration of the controller parameters.

In [96], the authors present a novel self-configuration model, based on software-defined networks (SDN) for time-sensitive networks. In existing configuration methods, end nodes send their data to a central management node. These methods require the manual configuration of the hosts. The proposed algorithm allows resources to be obtained in a transparent and automated manner, facilitating self-configuration in heterogeneous environments.

**Figure 8:**
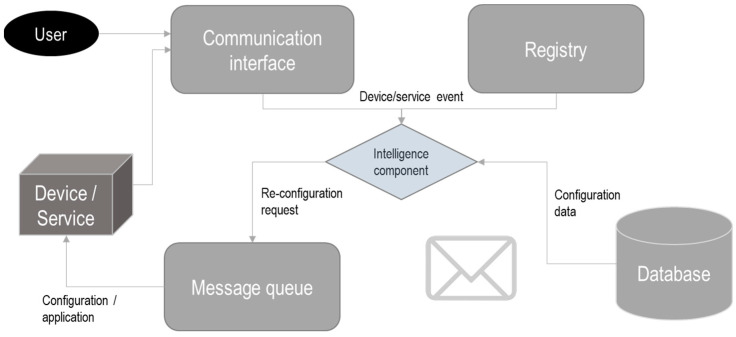
A self-configuration structure, as per enablers described in ASSIST-IoT. Source: the authors’ own diagram, based on [73].

In the ASSIST-IoT project, a self-configuration modular software (see Figure 8) was developed that allows heterogeneous services and devices to remain synchronized with their configurations. In addition, it allows to detect changes in the surrounding environment and update the configuration automatically, if necessary. On the other hand, the user can change the configuration manually and define configurations to be applied in cases of detecting a failure in a node [72,97].

Self-configuration is a characteristic that might bring multiple benefits to systems implementing the computing continuum. Nonetheless, research has focused on specific distributed systems (cloud-gaming, radiocommunications, etc., even within sensors that require calibration to properly function). Methodological approaches that drill down the process in various functional blocks also exist, opening the door to developments that could be applied to the heterogeneous node of the computing continuum.

#### 5.2.7. Self-Optimization

In 2003, IBM listed self-optimization as one of the four basic pillars of an autonomous system. IBM defined the concept of self-optimization in autonomous computing as the continuous improvement of the performance and efficiency of an autonomous system [43]. For Nami and Bertels [98], self-optimization is the ability of an autonomous system to allocate resources and use them in the most efficient way possible, meeting user requirements. In addition, they also state that autonomous system workload management and resource usage are two important points in self-optimization. Unlike self-scaling, self-optimization constantly monitors scaled resources to optimize their operation and performance. For the authors, the definition provided by IBM is more accurate when considering the field of the distributed computing continuum, as the horizontal resource allocation to maintain the quality of service (including acting in advance) is a competence of the self-scalability of nodes.

Zheng et al. [99] defined a model based on autonomous computing to automatically optimize services offered to users. When the system changes internally, that is, the parameters that influence the performance of the services provided to users change during its execution, dynamic self-optimization is executed. This improves the performance of the service to make it more efficient. When there are no big changes internally in the system, the static self-optimization prediction is executed. Both methods are combined to automatically improve the performance and efficiency of the services that the system provides to users. These are good principles that could be applied to the functioning of heterogeneous nodes of the continuum.

Moving away from the performance optimization of single elements, the authors of [100], propose a method to automatically optimize handover parameters for 5G networks. In these networks, the configuration of handover control parameter (HCP) settings is carried out manually or through self-optimization functions. Due to the large number of devices connected to the network, offering a stable connection over time has become one of the priorities in this type of network. Device handover occurs when a node moves between two cells of a network. The authors also classify the current algorithms as central optimization models, that is, the optimization is performed based on the performance of the network as a whole and not individually for each connected device. In [101], Sánchez-González et al. propose a rule-based self-optimization model for mobile networks that improves and speeds up convergence in the search for solutions. These rules are really information on how to solve specific problems. In addition, the authors state that this system has been fine-tuned to improve coverage and cell overlap within the same network.

Also rooted in network parameters optimization, in [102], the authors implement a self-optimization model for the nodes of cognitive wireless home networks, called the “Home Cognitive Resource Manager” (HCRM). The system uses several self-optimization algorithms and information captured from the execution environment in order to perform efficient radio resource management. To achieve its goal, the framework uses utility-based reasoning and compliance with policy regulations.

Trumler et al. [103] presented a model for creating self-organizing autonomous systems that are based on nodes located in the network. This system employs a mode of operation based on the hormonal system of humans. Each node sends information for self-organization through messages without using any extra communication system to avoid overloading the network. The objective of these messages is to know the consumption of the resources of the nodes to be able to optimize them in the most efficient way. The algorithm works in conjunction with a middleware also developed by the authors of the paper.

Looking at specific verticals of application, Wang et al. describe in the paper [104] an autonomous system for the self-optimization of the course of a ship. To do this, the objective to be achieved by the system is established, and, through various algorithms, it determines the most optimal and efficient control parameters of the ship’s course.

#### 5.2.8. Self-Adaptation

Self-adaptation is the self-* capability of the autonomous systems to adjust their behavior during execution in real-time. This adaptation is made to respond to changes in the perception of its environment and of the system itself [105,106].

Amiri et al. [107] propose an autonomous system that uses a dynamic router architecture capable of adapting at runtime. Several studies by the authors of the paper indicate that centralized routings offer greater reliability, while decentralized ones offer more performance. This system performs multi-criteria analysis to optimize and self-adapt the architecture between more centralized or more distributed routing to deliver the highest reliability and maximize performance.

The work described in [108] deals with the variation in the Particle Swarm Optimization (PSO) algorithm with dual self-adaptation and dual variation to improve the premature convergence problems of the standard version. The goal is to widen the search range for the optimal solution and improve the search accuracy, the algorithm’s rate of convergence, and its response speed. The authors affirm that, applied to the optimization of objective functions, their version of the PSO improves performance and results compared to the standard version.

Ardito [109] developed a system to self-adapt the operation of smartphone applications in real-time depending on the current battery consumption of the device. The goal is to reduce the energy consumption of smartphones and extend the life of their batteries. The method has several phases of operation. First, the power management module of the operating system obtains the consumption values through the hardware. Second, the module analyzes and divides the energy expenditure between each running application based on the current use of each one. Finally, it sends the information with a maximum threshold that must not be exceeded. If the application exceeds the threshold, the operating system sends it a warning to modify its operation, adapting itself according to its energy consumption.

In [110], Yuan et al. present a self-adaptive model called “CASC”, based on “MAPE” [56], to adapt the composition of services in real time. Self-adaptive composite services can automatically adjust in real time to changes in their surrounding environments. This system is capable of self-adapting by selecting new services or generating new schemes for the composition of the service.

While self-adaptation is a term used in wider communication environments (see above), applied to heterogeneous nodes in the continuum, self-adaptation would be the capacity to adapt the applications (containers, services, etc.) being run by such a node depending on the current execution of those (taking too long, consuming more resources than expected, requiring extra bandwidth, etc.) in runtime. Specifically related to this definition, the authors of [111] describe a multi-tier self-adaptation model for microservice systems that aims to improve the self-adaptation capabilities of microservice frameworks. In addition, they also present a self-adaptive description language with which to determine the adaptation logic at the different levels of microservice systems and a platform called “AdaptiveK8s” to provide support as a Kubernetes extension. The goal of all these efforts is to specify self-adaptation requirements at the different levels and to provide the necessary components to improve self-adaptation in microservice systems. Besides, Nallur and Bahsoon [112] propose a decentralized model in the cloud that uses heuristics so that service-based applications can self-adapt at runtime to the quality of service (QoS) requirements they offer to users.

Likewise, Boyapati and Szabo [113] developed a self-adaptive system for large-scale microservice architectures, based on “MAPE-K” [56]. The system is composed of two independent networks. In a network, the “MAPE-K” loop monitors the environment, analyzes the information received, and schedules tasks. On the other network, the scheduled tasks are executed in the managed system. All components are deployed on Docker and are related to each other by exposing REST APIs. The authors emphasize the use of open-source tools for the development and implementation of the proposed system.

In [114], the authors present a self-adaptive fog monitoring software that uses a hierarchical P2P architecture that is capable of modifying its operation based on an “MAPE-K” feedback loop. This variation in its behavior is possible thanks to the data that the system collects from its environment.

Self-adaptation in the literature implies the existence of various components (namely, services or microservices in modern distributed environments) whose execution can be modified in runtime to meet user requirements. The methodology “MAPE-K” seems to be widely employed in most practical implementations proposed in the found references.

#### 5.2.9. Self-Learning

Based on [115], self-learning is defined as the self-* capability of an autonomous system to improve its performance using unsupervised AI and ML over time. Although the usage of AI is applied to achieve other self-* capabilities (e.g., self-scaling), the exercise of valuing unlabeled historic data for self-improvement purposes can be considered a relevant capability by itself.

With the fast expansion of the IoT, a new concept called Internet of Vehicles (IoV) appeared. The calculations that these vehicles execute (with limited resources) are increasingly computationally expensive. To solve this, Vehicular Edge Computing (VEC) appeared, which allows vehicles to send these more expensive tasks to them. However, it is a problem that many vehicles compete for these computing resources at the same time. For this reason, Luo et al. [116] proposed a distributed computational offloading algorithm called “DISCO”, based on self-learning, where each vehicle gets its best offloading decision based on its information and the offloading decision of other vehicles. In [117], Srinivasan proposes a low-cost system for monitoring and predicting the status of the mechanical components of a car. To do this, through sensors installed in the vehicle, the necessary data are collected in real time. These data are sent to the cloud for processing using a self-learning algorithm, which predicts the future status of the monitored components. Finally, the analyzed information is sent to a mobile application so that it can be viewed by users.

In recent years, the use of drones has grown exponentially. They are used for military applications, agriculture, the analysis of aerial photographs, and even for civil use. In some of these more specific applications, drones need to perform operations that they cannot execute due to their limited resources. As a solution, the use of MEC nodes has been proposed to perform these calculations. Sacco et al. [118] developed a self-learning algorithm that allows the drone to decide whether to send the task to the MEC nodes using two different methodologies: time series and ML regressors. This decision is taken based on the predicted behavior of the drone.

In [119], Sudharsan et al. present an algorithm called “Train++” that allows for the training of ML models on IoT devices (such as sensors) with very limited resources. In this way, these types of components do not need to increase their performance and can dispense with ML model training services in the cloud to become intelligent self-learning devices at the edge of the network. Tam et al. [120], proposed a resource-optimized communication scheme for federated learning at the edge of the network. The objective is to perform classifications of images detected by remote IoT devices (sensors) in real time, using convolutional neural network algorithms. For this, a self-learning agent is used that communicates with the network orchestrator and the architecture to optimize the control of the resources of the IoT devices.

Shen et al. [121] present a self-learning algorithm for building energy management systems. This software uses the network formed by the IoT sensors (which should allow for calculations in the fog) to analyze, in a distributed way, the data obtained by the IoT sensors. The purpose of this system is to reduce the energy used, process the data from the sensors in the fog (instead of in the cloud), improve the comfort of the users, and increase the accuracy of the data predictions.

Some works have proposed the usage of self-learning to obtain ratings (positive, neutral, and negative) of status (e.g., comments of hotel reviews) [122], which could be very interesting in rating the capacity of a node to react to specific circumstances.

Considering that AI is one of the most researched fields in the current literature, the term self-learning presents difficulties in its association with heterogeneous nodes in the continuum. Only a few references express the need to use such capacities in computing elements in a network.

### 5.3. Literature Comparison

In this subsection, Table 1 is presented, comparing the works evaluated throughout the section. For each self-* capability, those articles related to each type of node capable of connecting to the computing continuum are reflected.

As can be seen in the table above, there are certain trends in the use of self-* capabilities depending on the spot on the continuum where they are applied. Self-configuration and self-scaling are the self-* capabilities that are most applied in all types of nodes described equally. Self-scaling and self-adaptation are the two self-* capabilities that are mostly applied in cloud servers and MEC nodes due to the importance of adapting and scaling the resources used to optimize them and reduce the energy consumption that these nodes need. Self-awareness and self-configuration are applied to servers and terminal nodes mainly due to the importance of knowing the environment that surrounds them and being able to reconfigure themselves appropriately based on changes in the environment. Finally, self-orchestration, self-diagnose, self-healing, and self-learning apply primarily to terminal nodes. This is because many of these nodes work together to achieve common goals. On the one hand, coordination is an important part in the organization of these nodes, as well as learning and predicting data through algorithms that collect information from the environment around them through sensors. On the other hand, the diagnosis and resolution of problems (or their prevention) is another important factor in this type of node for avoiding performance reductions in the networks they form due to their limited work capacity compared to large servers located in the cloud.

## 6. Future Research Directions

In the future, there will still be the need to deepen the coverage and deployment approaches of the selected self-* capabilities, identify and analyze new ones, and advance in the definition of standards that may allow for the creation of related open-source tools. The search for up-to-date use cases of monitoring tools should be enhanced, and surveys of experts in the field to find out their vision of the implementation and expansion of self-* capabilities today might be realized. Finally, following the reflection on this article’s limitation, the scope of the review should be expanded to include alternative sources such as open-source repositories or blogs. The authors of this paper look forward to performing such evolvements in future articles.

## 7. Conclusions

Over time, CC has led to new, more efficient, and more effective forms of computing: fog and edge computing. These offer advantages that the “cloud” is not capable of providing, such as lower energy consumption and better response times. This requires management by autonomous intelligent systems, which, apart from a holistic orchestration, might benefit from the implementation of self-* capabilities brought by heterogeneous computing nodes. Out of all the identified self-* capabilities, only a small group are really considered essential for those systems, and, as has been observed, there are hardly any references in the literature or systems that can be used as a basis for multiple solutions. The vast majority of the proposed solutions are customized systems that focus on very specific use cases (mostly distributed networks), which increases fragmentation and reduces the possibility of creating open standards and solid foundations that are valid for any field of application.

## Figures and Tables

**Table 1:** Evaluated works for each self-* capability and their spot in the continuum.

	Cloud Nodes	MEC Nodes	Edge Nodes	Far-Edge Nodes	Versatile Comp. Nodes	IoT Nodes
Self-awareness	[54,55,56,57,59]	[54,55,56,57,59]	[55,56,59]	[56,59]	[54,56,57,58,59]	[54,56,57,58,59]
Self-orchestration	[62]	[62]	[60,62,64]	[60,62,64]	[52,53]	[60,62,63,64]
Self-diagnose	[71,72,73]	[71,72,73]	[47,70,71,72,73]	[47,48,67,68,69,70,71,72,73]	[67,70,71,72,73]	[46,47,48,65,67,68,69,70,71,72,73]
Self-healing	[72,73,80]	[72,73,80]	[72,73,78,80]	[48,72,73,74,76,77,78,79,80]	[72,73,74,75,78,80]	[48,72,73,74,76,77,78,79,80]
Self-scaling	[72,82,83,84,85,86,87]	[72,82,83,84,85,86,87]	[72,86,87]	[72,86,87]	[72,86]	[72,86,87,88]
Self-configuration	[72,73,94,95,97]	[72,73,94,95,97]	[72,73,97]	[72,73,93,97]	[72,73,90,91,93,96,97]	[72,73,91,92,97]
Self-optimization	[99,102]	[99,102]	[100,101,102]	[100,101,102,104]	[100,101,102,103,104]	[100,101,102,104]
Self-adaptation	[107,110,111,112,113]	[107,110,111,112,113,114]		[48,114]	[109,114]	[48,114]
Self-learning	[117,119,122]	[116,118,121]	[49,116,120]	[49,116,117,118,119,120,121]	[117]	[49,116,117,118,119,120,121]

## Data Availability

Not applicable.
